# Temperature extremes and maternal health: differential risks of severe maternal morbidity during heatwaves and coldwaves in North Carolina

**DOI:** 10.1007/s00484-025-03079-z

**Published:** 2026-02-02

**Authors:** Sarah E. Ulrich, Maggie M. Sugg, Manan Roy, Jennifer D. Runkle

**Affiliations:** 1 Department of Geography and Planning, Appalachian State University, P.O. Box 32066, Boone, NC 28608 USA; 2 Department of Nutrition and Health Care Management, Beaver College of Health Sciences, Appalachian State University, 1179 State Farm Road, Boone, NC 28607 USA; 3 North Carolina Institute for Climate Studies, North Carolina State University, 151 Patton Avenue, Asheville, NC 28801 USA

**Keywords:** Severe maternal morbidity (SMM), Extreme temperatures, Matched analysis

## Abstract

Objective Climate change is increasingly recognized as a significant public health threat, particularly for vulnerable populations such as pregnant women. This study examines the association between heatwave and coldwave exposure and the risk of severe maternal morbidity (SMM) at delivery in North Carolina from 2011 to 2019. Methods We incorporate a matched analysis design using patient delivery data to identify disparities across the rural-urban continuum, measurements of neighborhood racial and economic inequality, geographic region, and sociodemographic groups (age, race/ethnicity, and insurance type). Results We found elevated risks of SMM associated with heatwave and coldwave exposures We observed an increased risk of severe maternal morbidity (SMM20) at delivery following exposure to heatwave (HW RR: 1.07, CI: 1.02 to 1.11) and coldwave (CW RR: 1.05, CI: 1.02 to 1.09) events during the last week of gestation in the advanced maternal age (> 35) subgroup. For coldwaves, the risk of SMM20 was elevated during the last week of gestation in both the western mountains (RR: 1.10, CI: 1.02 to 1.18) and rural (RR: 1.14, CI: 1.01 to 1.29) regions. Hispanic subgroups exhibited elevated SMM risks, particularly during heatwaves (lag0 to lag7, RR: 1.09, CI: 1.00 to 1.18) and coldwaves (lag0 to lag3, RR: 1.10, CI: 1.01 to 1.19; lag0 to lag7, RR: 1.04, CI: 1.00 to 1.07). Conclusion Our findings suggest that extreme temperature events exacerbate vulnerabilities in specific maternal subpopulations, underscoring the need to account for local context and population characteristics in maternal health assessments. Future research should continue to explore these associations and the role of additional environmental and social factors in SMM risk.

**Supplementary Information:**

The online version contains supplementary material available at 10.1007/s00484-025-03079-z.

## Introduction

Severe maternal morbidity (SMM) refers to unexpected, life-threatening complications during labor and delivery that are considered near-misses for maternal mortality. Despite advancements in prenatal care, SMM rates have risen significantly, increasing from 146.8 to 179.8 per 10,000 hospital deliveries between 2008 and 2021 (Fink et al. 2023). Currently, SMM affects approximately one in every 69 pregnancies, and women with SMM during hospital deliveries are over 10 times more likely to be readmitted postpartum (Black et al.,^ 2021^). For every maternal death, there are 70 cases of SMM (Matas et al., 2021), with 20 to 30 individuals enduring significant lifelong complications that adversely affect their health and well-being (Fink et al., 2023). While maternal mortality represents the most severe pregnancy-related outcome, the more prevalent maternal morbidities—often near-death experiences—serve as critical warning signs. Identifying the risk factors for SMM is essential for developing interventions to prevent progression to mortality.

Rising SMM rates are particularly pronounced among racial and ethnic minorities, women of advanced maternal age, and those with comorbid conditions (Fink et al. 2023; Minehart et al. 2021; Liese et al. 2019). Black women, in particular, experience the highest proportion of SMM across all pregnancy intervals, with up to 70% greater risk during the antepartum period (Liese et al., 2019). Maternal characteristics such as low socioeconomic status, obesity, and medical interventions, including cesarean delivery, contribute to the rising prevalence of SMM. However, the role of environmental and structural factors remains understudied. Given that up to 44% of SMM cases may be preventable (Matas et al., 2021), understanding these broader influences is a critical research priority with significant implications for maternal healthcare delivery.

The rise in SMM among US women is occurring against the backdrop of increasing extreme temperatures due to climate change, which is projected to intensify both nighttime and daytime temperatures (Liu et al., 2024)(. While substantial evidence links extreme temperatures to maternal health risks, such as gestational diabetes (Baharav et al. 2023; Qu et al. 2021), preterm birth (Baharav et al. 2023; Chersich et al. 2023; Rekha et al., n.d.; Beltran, Wu, and Laurent 2014; Kuehn and McCormick 2017), hypertensive disorders of pregnancy (Baharav et al. 2023; Beltran, Wu, and Laurent 2014; Cil and Cameron 2017; Qu et al. 2021), and maternal mental health conditions (Baharav et al. 2023; Ulrich et al. 2025; Runkle et al. 2024), few studies have investigated climate-sensitive exposures as risk factors for SMM. However, recent research has identified extreme heat exposure as a significant contributor to increased SMM risk (Harden, Runkle, and Sugg 2022; Jiao et al. 2023).

This study examines the relationship between extreme temperature events—specifically heatwaves and coldwaves—and SMM in North Carolina, a Southeastern U.S. state with a subtropical climate and elevated maternal mortality rates. Additionally, it explores how structural factors, such as systemic racism, economic inequality, geographic disparities, and rurality, influence and potentially amplify the effects of extreme temperatures on SMM. By analyzing these environmental and contextual interactions, this research aims to shed light on preventable risk factors and advance strategies mitigating maternal health disparities.

##  Methods & data

### SMM-related hospital deliveries

SMM data were obtained from inpatient hospital records. Our sample comprised 13,851 daily deliveries complicated by SMM in North Carolina from 2011 to 2019.

### Outcome definition

The Centers for Disease Control and Prevention (CDC) defines severe maternal morbidity (SMM) based on a composite of twenty-one procedures and diagnostic indicators derived from the ninth and tenth revisions of the International Classification of Diseases codes (ICD-9 and ICD-10). We operationalized SMM into two primary outcomes: (1) SMM including blood transfusion (SMM21) and (2) SMM excluding blood transfusion (SMM20). Blood transfusion is the most commonly occurring indicator of SMM (Callaghan, Creanga, and Kuklina 2012; Creanga et al. 2014); however, ICD-9 or ICD-10 procedure codes do not specify the number of units transfused, potentially leading to an overestimation of SMM21 rates. To address this limitation, prior studies have often focused on SMM20 (Harden et al., 2022; Oakley et al., 2023; Snowden et al., 2021). Following this precedent, our primary analysis centers on SMM20 as the primary outcome, with SMM21 included in supplemental analyses. The specific codes used to define SMM20 and SMM21 are listed in Supplemental Table 1.

While our binary classification (SMM with/without transfusion) follows established precedent in the literature, we acknowledge substantial clinical heterogeneity within these categories. SMM21 (with transfusion) may capture a broader range of severity, including less severe hemorrhagic events, whereas SMM20 (without transfusion) encompasses rarer but often more serious complications such as stroke, sepsis, cardiac arrest, acute renal failure, and eclampsia. Our focus on SMM20 as the primary outcome reflects an emphasis on these potentially life-threatening non-hemorrhagic complications.

### Heatwave and Coldwave calculations

We defined heatwave and coldwave events using the excess heat factor (EHF) and excess cold factor (ECF), respectively. These indices were calculated at the zip code tabulation area (ZCTA) level using daily average temperature (Tavg) data (Nairn and Fawcett 2015; Wang et al. 2016). EHF and ECF quantify the intensity of extreme temperature events over a consecutive three-day period exceeding the 95th percentile of Tavg based on local climatology and accounts for a prior 30-day acclimatization period (Scalley et al., 2015). This acclimatization period considers physiological adaptations to temperature fluctuations, influencing population-level responses to extreme heat and cold events (Scalley et al., 2015). By calculating heatwave and coldwave exposure at the ZCTA level, our approach captures localized variations in extreme temperature exposure and climate across North Carolina’s diverse geography, which varies significantly from the western mountainous to the eastern coastal region.

### Covariates

We considered both individual-level and community-level factors as potential modifiers of the relationship between extreme temperatures and SMM. Individual-level covariates included: advanced maternal age (above or below 35), race (Black, White, or Other), ethnicity (Hispanic or Not Hispanic), and insurance type (Private, Medicaid, Self-Pay, or Other). These variables have been identified as key risk factors in prior research (Smith and Hardeman 2020; Huang et al. 2021; Chersich et al. 2020; Ngo and Horton 2016; Basu, Sarovar, and Malig 2016; Jiao et al. 2023). Socioeconomic status was assessed using insurance type (i.e., Medicaid enrollment) (Qu et al. 2021). Trimester of exposure was defined using ICD codes as outlined in Supplemental Table 1.

Community-level covariates considered in our study included: residential segregation (a proxy of structural racism), economic segregation, rural-urban status, and physiographic climatic region (Western Mountains, Piedmont, and Coastal Plain). Rural-Urban Commuting Area (RUCA) codes were used to assess variations in SMM risk along the rural-urban continuum (Sugg, Konrad, and Fuhrmann 2016; Ulrich et al.^2023^). RUCA scores were calculated at the ZCTA level and aggregated into rural (1–3), suburban (4–6), and urban (7–10) (“USDA ERS - Rural-Urban Commuting Area Codes,” 2020).

The Index of Concentration at the Extremes calculated from 2014 to 2018 American Community Survey 5-year estimates of household income, race, and ethnicity data to quantify measurements of structural racism and economic inequality at the ZCTA level (Massey 1996; Krieger et al. 2016). Index scores range from − 1 to 1, where − 1 represents communities with extreme concentration of disadvantage (either economic or racial), 0 indicates balanced communities, and 1 represents extreme concentration of advantage. We computed two Index of Concentration at the Extremes measures: Economic segregation (ICE-E) and residential racial segregation (ICE-R).

The economic segregation index was derived by assessing the spatial concentration of economic privilege and deprivation within each ZCTA. We categorized communities into tertiles: T1 represented majority low-income communities (highest concentration of households in the lowest income bracket), T2 represented communities with moderate income distribution, and T3 represented majority high-income households (highest concentration of households in the upper income bracket).

The residential racial segregation index was calculated using racial composition at the ZCTA level. T1 represented majority Black communities, T2 represented communities with a more racially mixed composition, and T3 represented majority White communities. These Index of Concentration at the Extremes measures allowed us to account for both economic and racial structural inequities in the assessment of maternal health outcomes, providing a more granular analysis of how these factors may modify the relationship between extreme temperatures and SMM.

### Matched analysis

We conducted a matched analysis to compare maternal outcomes during heatwaves and coldwaves with outcomes during unexposed periods, following established methods in environmental health research (Sun et al. 2020; Bobb et al. 2014; Liu et al. 2017; Yan et al. 2021). Heatwaves (May to September) and coldwaves (October to April) were matched to non-exposed periods within the same ZCTA. Each extreme temperature event (including the two days prior and the seven days following) was matched to three randomly selected unexposed periods, ensuring that these periods did not overlap with other extreme temperature events (Sun et al. 2020; Liu et al. 2017). This approach minimized seasonal confounding effects and allowed for more precise estimates of temperature-related risks.

We restricted our analysis to ZCTAs with complete data and at least one documented heatwave or coldwave during the study period. We aggregated individual-level SMM20 and SMM21 cases to the ZCTA level for each date in the study period (May to September 2011 to 2019 for heatwaves; October to April 2011 to 2019 for coldwaves).

### Statistical analysis

Generalized nonlinear regression model estimated the relative risk (RR) of SMM during extreme temperature events compared to matched non-exposed periods, incorporating a distributed lag model (DLM) for heatwave and coldwave exposure (Bobb et al. 2014). RR were assessed for each day within the heatwave or coldwave periods, with cumulative risk estimated across (1) *acute exposure*: lag0 to lag3 and (2) *cumulative exposure*: lag0 to lag7. To examine effect modification, analyses were stratified by subpopulations based on race, ethnicity, advanced maternal age, and Medicaid enrollment, as well as geographic factors such as ICE metrics, rural-urban status, and physiographic regions. Separate regression models were fitted for each subgroup within each covariate category (e.g., age above 35, age below 35, Hispanic ethnicity, Black race, Medicaid insurance, etc.) and for each exposure period (lag0 to lag3 and lag0 to lag7), for both heatwaves and coldwaves. Each model was estimated using a stratified sample restricted to the subgroup of interest, such that relative risks represent comparisons between exposure (heatwave or coldwave) and non-exposure periods within that subgroup rather than across subgroups. Because models were stratified by covariate subgroup, relative risks represent comparisons between heatwave (or coldwave) and non-heatwave (or non-coldwave) periods within each subgroup. All statistical analyses were performed in R using the “dlnm” and “lme4” packages. Statistical significance was defined as a 2-sided p-value < 0.05.

## Results

**Fig. 1:**
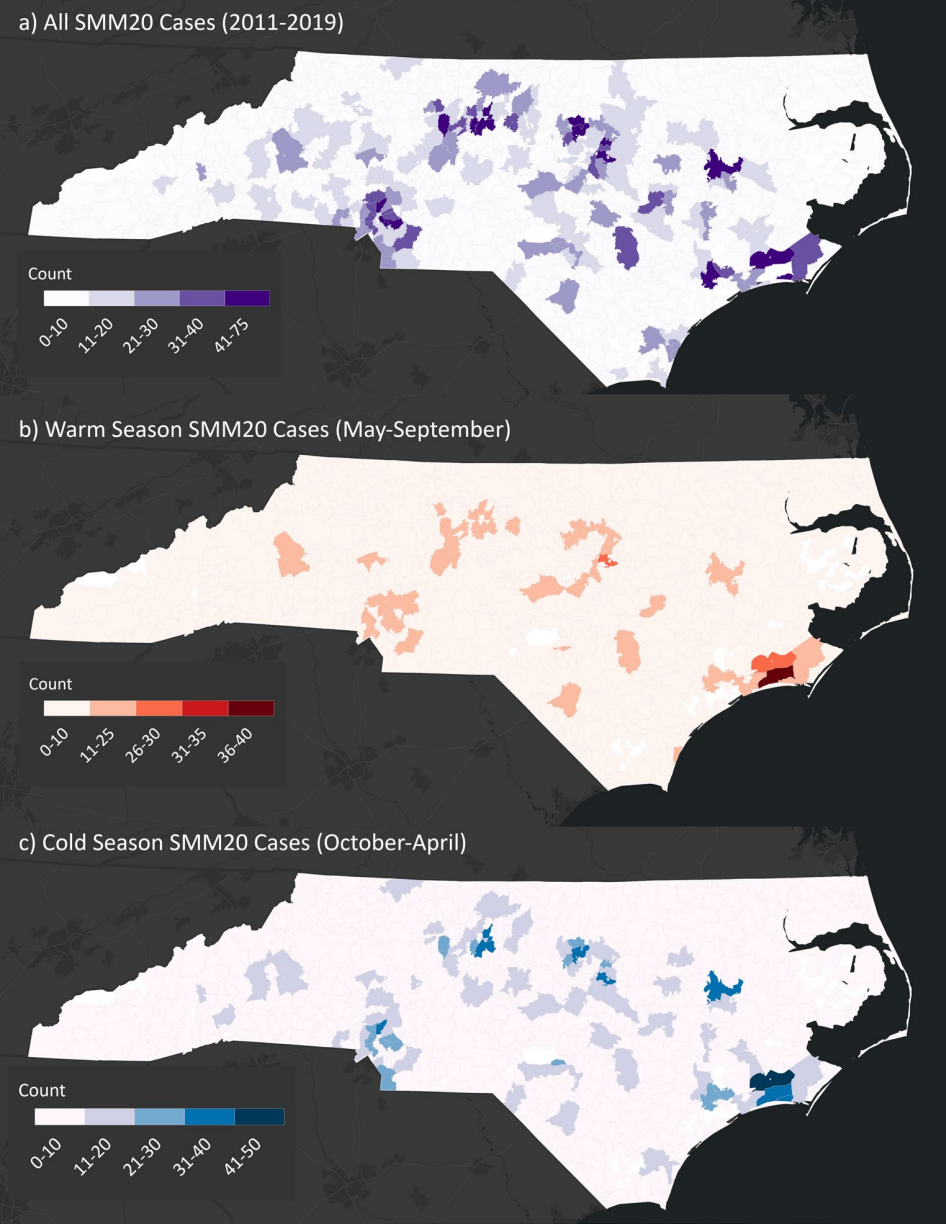
The total number of cases of severe maternal morbidity without blood transfusion (SMM20) for each ZCTA during the study period (2011 to 2019), warm season (May to September 2011–2019), and cold season (October to April 2011 to 2019)

SMM20 case counts were mapped for each ZCTA during the study period, categorized by warm and cold season (Fig. 1) and for heatwave and coldwave days (Supplemental Fig. 1). SMM21 case counts are mapped in Supplemental Fig. 4.

Table 1 presents the distribution of SMM20 cases by season, along with corresponding sociodemographic characteristics and regional factors. Our analysis revealed 2,421 SMM20 cases during the warm season (May-September) and 3,332 cases during the cold season (October-April). Notable demographic differences included a higher proportion of advanced maternal age cases during cold seasons (21.3%) compared to warm seasons (20.1%). Among racial groups, Black women accounted for 36.4% of warm season and 33.2% of cold season cases. Hispanic women represented 11.5% of warm season cases versus 10.3% in cold season. Insurance coverage distribution showed that Medicaid covered 50.2% of warm season and 49.6% of cold season cases. Geographically, the Piedmont region had the highest concentration of cases in both seasons (52.8% warm, 53.1% cold), followed by the Coastal region (34.1% warm, 35.7% cold) and Western mountains (13.1% warm, 11.1% cold).

**Table 1:** Case counts and demographic variables for severe maternal morbidity without blood transfusion (SMM20) deliveries in North Carolina from 2011 to 2019, stratified by warm (May to September) and cold (October to April) seasons

	level	All Deliveries *n* (%)	Warm Season (May-September) *n* (%)	Cold Season (October-April) *n* (%)
Total		5753	2421	3332
Age group	18–35	4516 (78.4)	1914 (79.0)	2602 (78.0)
	35+	1195 (20.8)	487 (20.1)	708 (21.3)
	Missing/Unknown	42 (0.7)	20 (0.8)	22 (0.7)
Race	Black	1914 (33.3)	883 (36.4)	1105 (33.2)
	White	2729 (47.4)	1136 (46.9)	1593 (47.8)
	Other	943 (16.4)	402 (16.6)	541 (16.2)
	Missing/Unknown	167 (2.9)	74 (3.1)	93 (2.8)
Ethnicity	Hispanic	623 (10.8)	279 (11.5)	344 (10.3)
	Not Hispanic	4944 (85.9)	2069 (85.5)	2875 (86.3)
	Unknown	186 (3.2)	73 (3.0)	113 (3.4)
Insurance	Private/Commercial	2354 (40.9)	988 (40.8)	1366 (41.0)
	Medicaid	2876 (50.0)	1215 (50.2)	1661 (49.8)
	Self-Pay	110 (1.9)	44 (1.8)	66 (2.0)
	Other	389 (6.8)	162 (6.7)	227 (6.8)
	Missing/ Unknown	24 (0.4)	12 (0.5)	12 (0.4)
Economic Segregation (ICE-E)	Tertile 1: Low (majority low-income)	1196 (20.9)	511 (21.2)	685 (20.7)
	Tertile 2: Mixed-income	2229 (39.0)	950 (39.5)	1279 (38.7)
	Tertile 3: High (majority high-income)	2284 (40.0)	944 (39.3)	1340 (40.6)
Residential Racial Segregation (ICE-R)	Tertile 1: Low (majority Black)	2538 (44.5)	1050 (43.7)	1488 (45.0)
	Tertile 2:Mid	2282 (40.0)	962 (40.0)	1320 (40.0)
	Tertile 3:High (majority white)	889 (15.6)	393 (16.3)	496 (15.0)
Urban-rural	Rural	361 (6.3)	162 (6.7)	199 (6.0)
	Suburban	977 (17.1)	417 (17.3)	560 (16.9)
	Urban	4371 (76.6)	1826 (75.9)	2545 (77.0)
Physiographic Region	Western Mountains	682 (11.9)	314 (13.1)	368 (11.1)
	Piedmont	3027 (53.0)	1271 (52.8)	1756 (53.1)
	Coastal Plain	2000 (35.0)	820 (34.1)	1180 (35.7)

*ICE *Index of Concentration of Extremes

The distribution of residential racial and economic segregation tertiles were mapped in Supplemental Fig. 2. Predominantly low-income communities were concentrated in the state’s eastern coastal and western mountainous regions, which also tend to be more rural. Predominantly minority (e.g., Black and Hispanic) communities were primarily located in the northeastern coastal region, more generally, in the eastern region of the state. The distribution of rural and urban areas across NC was illustrated in Supplemental Fig. 3.

**Fig. 2:**
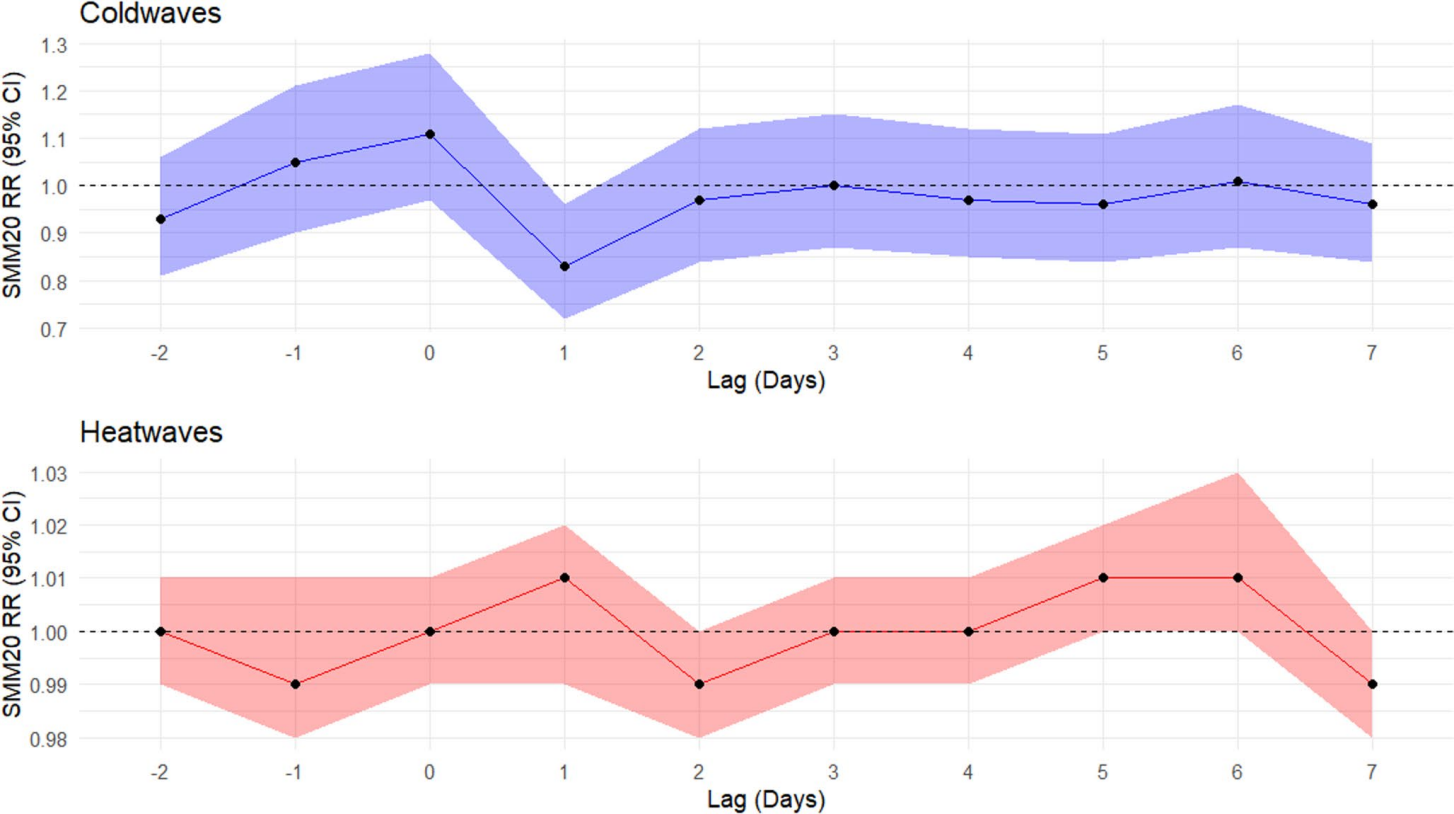
Daily relative risk (RR) and 95% confidence interval (CI) values for SMM20 during heatwave and coldwave periods (lag-2 to lag7)

Case counts by subgroup for SMM20 during heatwave and coldwave periods and matched unexposed periods are listed in Supplemental Table 2. Daily lag results for heatwaves and coldwaves are illustrated in Fig. 2 and confidence interval values are provided in Supplemental Table 3. Daily lag results did not indicate elevated SMM20 risks on heatwave for the same day of exposure (lag0) or any of the single-day lags (lag1 to lag7). Coldwave exposure on lag day 1 was protective (RR: 0.83, 95%CI: 0.72, 0.96).

**Fig. 3:**
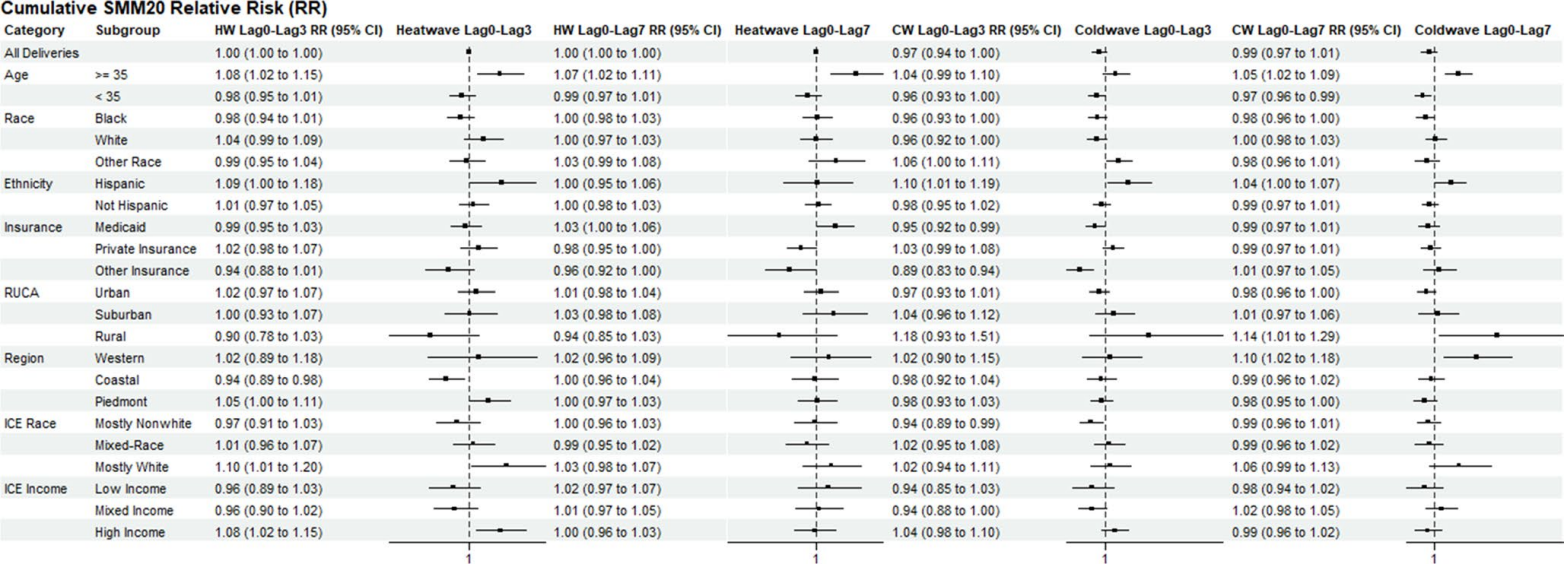
Cumulative relative risk (RR) values for SMM20 for subgroups during lag0 to lag3 and lag0 to lag7 heatwave and coldwave periods.Separate models were fitted for each covariate subgroup and exposure period. Each relative risk estimate represents an independent model comparing heatwave/coldwave periods to matched non-exposed periods within that specific subgroup

Model results forhe acute and cumulative effects of SMM20 for heatwave and coldwave periods (lag0 to lag3 and lag0 to lag7) are plotted in Fig. 3. The self-pay insurance subgroup was omitted from our results due to a low sample size for cases during heatwave and coldwave periods. Cumulative relative risks for lag0 to lag3 and lag0 to lag7 periods indicated elevated SMM20 risks for specific subgroups. We observed an increased risk of SMM20 at delivery following exposure to heatwave (HW RR: 1.07, CI: 1.02 to 1.11) or coldwave (CW RR: 1.05, CI: 1.02 to 1.09) events during the last week of gestation in the advanced maternal age (> 35) subgroup. The elevated risk of SMM20 for advanced maternal age persisted in the acute (Lag0-Lag3) and cumulative (lag0- lag7) exposure periods and the days immediately following (Age > 35 RR: 1.08, CI: 1.02 to 1.15). An elevated SMM20 risk was observed during the lag0-3 heatwave period (RR: 1.05; CI: 1.00 to 1.11), though not statistically significant. For coldwaves, SMM20 risk at delivery was elevated during the last week of gestation in western mountains (RR: 1.10, CI: 1.02 to 1.18) and rural (RR: 1.14, CI: 1.01 to 1.29) regions. Elevated risks for SMM20 were also noted during lag0 to lag3 heatwave periods in majority white (RR_ICE-R_: 1.10, CI: 1.01 to 1.20) and majority high-income (RR_ICE-E_: 1.08, CI: 1.02 to 1.15) neighborhoods.

Conversely, SMM20 risk following lag0 to lag3 coldwave periods was lower in Medicaid (RR: 0.95, CI: 0.92 to 0.99) and Other insurance groups (RR: 0.89, CI: 0.83 to 0.94), as well as in predominantly nonwhite neighborhoods (RR: 0.94, CI: 0.89 to 0.99). Elevated SMM20 relative risk values were observed in Hispanic subgroups during lag0 to lag7 heatwave periods (RR: 1.09, CI: 1.00 to 1.18) and during both lag0 to lag3 (RR: 1.10, CI: 1.01 to 1.19) and lag0 to lag7 (RR: 1.04, CI: 1.00 to 1.07) coldwave periods.

As a sensitivity analysis, we examined the daily and cumulative relative risks for SMM21 (Supplemental Figs. 5 and 6, respectively). The risk of SMM21 at delivery was higher on the same day of a heatwave event (lag0) (RR: 1.12, CI: 1.01 to 1.25). Additionally, we observed an increased risk on the third day following a coldwave event (lag3) (RR: 1.13, CI: 1.02 to 1.24), suggesting potential delayed effects of extreme cold exposure.

## Discussion

This analysis aims to identify the association between maternal exposure to extreme heat and cold, the risk of severe maternal morbidity (SMM) at delivery, and key individual and community-level determinants. Our findings highlight the growing threat of extreme temperatures in the southeastern U.S.and the disproportionate impact on perinatal populations. By incorporating rurality, geographic region, and neighborhood socio-demographic context, we provide critical insights into how climate-related stressors intersect with maternal health vulnerabilities, particularly among advanced maternal age subgroups. The observed regional and demographic differences emphasize the need to consider local context and population characteristics when assessing the maternal health impacts of climate change. Women of advanced maternal age may be more sensitive to thermal extremes due to age-related physiological changes, such as reduced cardiovascular and thermoregulatory resilience, which can amplify vulnerability to heat and cold stress. Additionally, predominantly rural regions may be particularly vulnerable due to limited access to obstetric care, longer travel distances to healthcare facilities, fewer specialized maternal health providers, and a higher prevalence of structural determinants of health inequities, such as poverty or inadequate housing, which can increase exposure to extreme temperatures. These intersecting risk factors underscore the need for targeted interventions that address both environmental and healthcare disparities.

Multiple pathways support the biological plausibility of temperature extremes affecting SMM20 (without blood transfusion) outcomes. Heatwave exposure may trigger acute physiologic stress that exacerbates underlying cardiac or vascular vulnerabilities through mechanisms including dehydration, increased cardiac workload, endothelial dysfunction, or inflammation (Basu et al. 2002; Samuels et al. 2022) Similarly, coldwave exposure may increase risks through vasoconstriction, elevated blood pressure, increased blood viscosity, or prothrombotic states (Analitis et al. 2008; Khosravipour & Golbabaei, 2024). Pregnant individuals may be particularly vulnerable to these mechanisms due to pregnancy-related physiologic changes, including increased blood volume, altered cardiac output, and changes in thermoregulation.

Our results suggest extreme temperature events may exacerbate existing risks, particularly among older mothers. Consistent with prior research, we identified an increased risk of SMM associated with heatwave exposure during the last week of gestation, aligning with findings that effects are modified by education level and season of conception (Jiao et al., 2023). Using a large dataset of deliveries in North Carolina, we identified an association between exposure to extreme temperatures during the final week of gestation and an elevated risk of SMM at delivery in specific subpopulations and subregions.

Regional disparities were also evident. The Piedmont region exhibited heightened SMM20 risks during heatwaves, whereas western and rural regions showed increased risks during coldwaves. Previous studies have documented higher SMM rates in both the most urban and most rural counties, with racial disparities shaping these patterns (Kozhimannil et al. 2019; Luke et al. 2021). Even after adjusting for sociodemographic and clinical factors, rural residents face a 9% greater likelihood of SMM and mortality than urban residents (Kozhimannil et al. 2019). Our study is the first to show increased SMM risks in rural areas during cold waves, emphasizing the need for targeted interventions addressing clinical shortages and broader social determinants of health.

Our findings of elevated SMM risk during acute heatwave periods in high-income and majority-white areas may reflect regional patterns in North Carolina, where the western region is predominantly white but also has lower average income levels. These underlying geographic and sociodemographic patterns likely contribute to the observed associations. Overall, the results suggest that structural advantage (e.g., income, whiteness) may not be fully protective against the adverse maternal health impacts of extreme heat exposure.

Prior research has shown that Hispanic women may be at increased risk for SMM compared to non-Hispanic white women (Creanga et al., 2010); while other studies have shown a lower risk among Hispanic women (Liese et al., 2019; Admon et al., 2018). In our study, Hispanic women consistently displayed elevated risks of SMM20 during both heatwave and coldwave periods. Reasons for this might include variations in hospital quality, institutionalized racism in the healthcare system (Leonard et al., 2019), and increased vulnerability to exposure to heatwaves or coldwaves (Liu et al., 2024).

Occupational and housing factors likely contribute to this disparity. Occupational factors, such as working outdoors in agriculture or construction, are particularly relevant, as these jobs expose individuals to prolonged heat or cold exposure, potentially increasing the risk of thermal stress and related complications (Flocks et al. 2013; Mirabelli et al. 2010; Gubernot et al. 2014). Many Hispanic women work in low-wage, physically demanding jobs that lack sufficient environmental climatic control or flexibility to manage heat stress, making them more vulnerable to extreme temperatures (Wildsmith et al. 2018; ToPoel et al. 2017; Ross and Bateman 2019). Additionally, poor housing conditions may exacerbate the risk associated with extreme weather events. Hispanic women are disproportionately represented in substandard housing, which may lack proper insulation or climate control like air conditioning or heating, leading to increased exposure to extreme heat or cold (Aiken et al. 2021; Cook et al. 1994; Newman et al. 2021; Scott et al. 2025). These multifaceted stressors highlight the importance of considering structural and environmental determinants of health in understanding the heightened maternal health risks among Hispanic women during extreme temperature events.

Lastly, results from sensitivity analysis revealed an attenuated association between same-day heatwave exposure and an elevated risk of SMM21. However, this result may be influenced by including blood transfusions in the SMM21 definition, warranting further investigation. Heat wave exposure can lead to dehydration, reducing plasma volume and increasing blood viscosity, which may impair uteroplacental blood flow and elevate the risk of placental abruption and postpartum hemorrhage (Baharave et al. 2023, Abrams et al. 2011). Elevated risks of SMM following heatwave and coldwave exposure was evident, suggesting that extreme temperatures may contribute to a broader range of life-threatening pregnancy complications beyond hemorrhage. Heatwave exposure has been associated with an increased risk of hypertensive disorders in pregnancy (Mao et al. 2023), organ dysfunction (Varghese et al. 2005; Pease et al. 2009), and cardiac complications (Liu et al. 2022), which can escalate to conditions such as eclampsia, stroke, or acute kidney injury, all of which are markers of severe maternal morbidity. Similarly, exposure to coldwaves may heighten the risk of thromboembolic events, such as deep vein thrombosis or pulmonary embolism, as cold stress can lead to vasoconstriction, increased blood viscosity, and systemic inflammation (Liu et al. 2015; Hess et al. 2009; Styler et al. 2022; Zhao et al. 2020). Additionally, both thermal extremes may exacerbate preexisting conditions, including gestational diabetes and cardiovascular disease (Fares 2013; Mercer 2003), which can further increase the likelihood of severe complications during delivery. The persistence of elevated SMM risk without transfusions highlights the multifaceted ways in which climate-related exposures can impact maternal health, underscoring the need for further research and targeted interventions to mitigate these risks..

An important limitation of the current evidence base, including our study, is its observational nature. While epidemiological studies can identify associations between temperature extremes and adverse maternal outcomes, experimental research is needed to establish safe thresholds of heat and cold exposure during pregnancy and to elucidate the underlying physiological mechanisms (Samuels et al., 2022). Emerging experimental work in fields like thermal ergonomics laboratories is needed to define safe limits for heat exposure during pregnancy through controlled trials, which will be essential for developing evidence-based clinical guidelines and integrating temperature-related risk assessments into prenatal care protocols.

In the context of women’s health, this study highlights the intersection of climate extremes and maternal morbidity, underscoring the need to better understand how environmental factors, such as heatwaves or coldwaves, exacerbate existing vulnerabilities in maternal populations. Women of advanced maternal age, those living in rural areas, and Hispanic women, in particular, appear to face increased risks of SMM during extreme temperature events. Integrating climate resilience into women’s healthcare frameworks, including prenatal and perinatal care, will be vital to reducing the health burdens of climate change. For healthcare providers, our findings emphasize the importance of assessing environmental risk factors and implementing temperature-specific protocols for high-risk pregnancies, especially during extreme weather events.

### Strengths and limitations

The strengths of our study include the use of a matched analysis method that accounts for robust comparisons of SMM outcomes during heatwave and coldwave periods. This approach also accounts for sub-regional acclimatization at the ZCTA level. The matched analysis approach eliminates confounding introduced by factors that vary across strata by contrasting within matched strata (Yan et al., 2023; Armstrong et al., 2014). Additionally, our study benefits from a nine-year comprehensive dataset (2011–2019), providing sufficient statistical power to detect associations in subgroup analyses. Including both extreme heat and extreme cold exposures in the same study framework allows for a direct comparison of their effects on maternal health outcomes. Our use of localized climate metrics (EHF and ECF) incorporating acclimatization provides a more physiologically relevant measure of thermal stress than absolute temperature thresholds.

Our study’s methodological approach offers several advantages. By stratifying the analysis by important sociodemographic and geographic factors, we identified vulnerable subpopulations that might otherwise be overlooked in aggregate analyses. The inclusion of structural factors like residential racial and economic segregation (measured by the Index of Concentration at the Extremes) provides important context for understanding how social determinants interact with environmental exposures, moving beyond individual-level risk factors to consider community-level influences on maternal health outcomes.

Our study is limited by the assumption that all residents within a ZCTA are uniformly exposed to heatwave or coldwave days. Our sample also did not include data on psychiatric medications, which can interact with extreme temperature exposure. Other important clinical data limitations include the absence of information on pre-pregnancy comorbidities, pregnancy complications prior to delivery, and detailed clinical management during delivery hospitalization. Our reliance on ICD-9 and ICD-10 codes for SMM identification may have resulted in some misclassification, as these codes are primarily designed for billing rather than research purposes. Our finding of elevated acute heatwave-related SMM risk in high-income and majority-white areas was unexpected and may reflect limitations in our exposure assessment (assuming uniform exposure within ZCTAs), outcome measurement (potential detection bias related to healthcare access), or unmeasured confounding. We lacked ZCTA-level relative humidity data, which may result in exposure misclassification for some pregnant women.

The geographic focus on North Carolina, while allowing for detailed regional analysis, may limit the generalizability of our findings to regions with different climatic conditions, healthcare systems, or demographic compositions. The observed associations may differ in areas with different baseline climate conditions or population characteristics. Future studies should include maternal populations outside North Carolina and directly measure access to protective resources to confirm the generalizability of our findings.

## Conclusion

This study highlights the significant relationship between maternal exposure to extreme temperatures and the risk of severe maternal morbidity at delivery, emphasizing the importance of considering individual and community-level determinants. Our findings suggest that both heatwaves and coldwaves may exacerbate existing maternal health vulnerabilities, particularly among women of advanced maternal age, Hispanic women, and those living in rural areas. The observed regional and demographic disparities underscore the need for targeted interventions that address both environmental and structural disparities in maternal healthcare, with a first step involving raised awareness of temperature sensitivities in prenatal care visits. Future research should further investigate the mechanisms underlying these associations, including the role of preexisting health conditions, healthcare access, and broader environmental exposures such as air pollution. Additionally, efforts to identify protective factors, including social and community-based resilience strategies, will be essential in mitigating climate-related maternal health risks. Future research should further investigate the mechanisms underlying these associations, including the role of preexisting health conditions, healthcare access, and broader environmental exposures such as air pollution. Healthcare systems and policymakers should incorporate these findings into climate adaptation strategies, particularly focusing on enhancing maternal healthcare infrastructure in vulnerable communities and developing temperature-specific protocols for high-risk pregnancies.

## Supplementary Information

Below is the link to the electronic supplementary material.

Supplementary file 1 Supplemental Figure 1. Count of heatwave and coldwave days by ZCTA (JPG 734 KB)

Supplementary file 2 Supplemental Figure 2. Economic segregation (ICE-E) and residential racial segregation (ICER) tertiles at the ZCTA level in North Carolina using data from the 2018 American Community Survey with 5-year estimates (PNG 1.76 MB)

Supplementary file 3 Supplemental Figure 3. Distribution of Rural-Urban Commuting Area (RUCA) codes at the 2010 zip code tabulation area (ZCTA) level for North Carolina (PNG 1.25 MB)

Supplementary file 4 Supplemental Figure 4. The total number of cases of severe maternal morbidity with blood transfusion (SMM21) for each ZCTA during the study period (2011 to 2019), warm season (May to September, 2011-2019), and cold season (October to April, 2011 to 2019) (PNG 2.41 MB)

Supplementary file 5 Supplemental Figure 5. Daily relative risk values for SMM21 during heatwave and coldwave days (PNG 6.46 KB)

Supplementary file 6 Supplemental Figure 6. Cumulative relative risk (RR) values for SMM21 during lag0 to lag3 and lag0 to lag7 heatwave and coldwave periods (PNG 28.2 KB)

Supplementary file 7 (PDF 6.38 MB)
