# Supplementary figures and images for: Temperature extremes and maternal health: differential risks of severe maternal morbidity during heatwaves and coldwaves in North Carolina
Source: Int J Biometeorol. 2026 Feb 2;70(2):45. doi: 10.1007/s00484-025-03079-z (PMC12864237; doi:10.1007/s00484-025-03079-z)

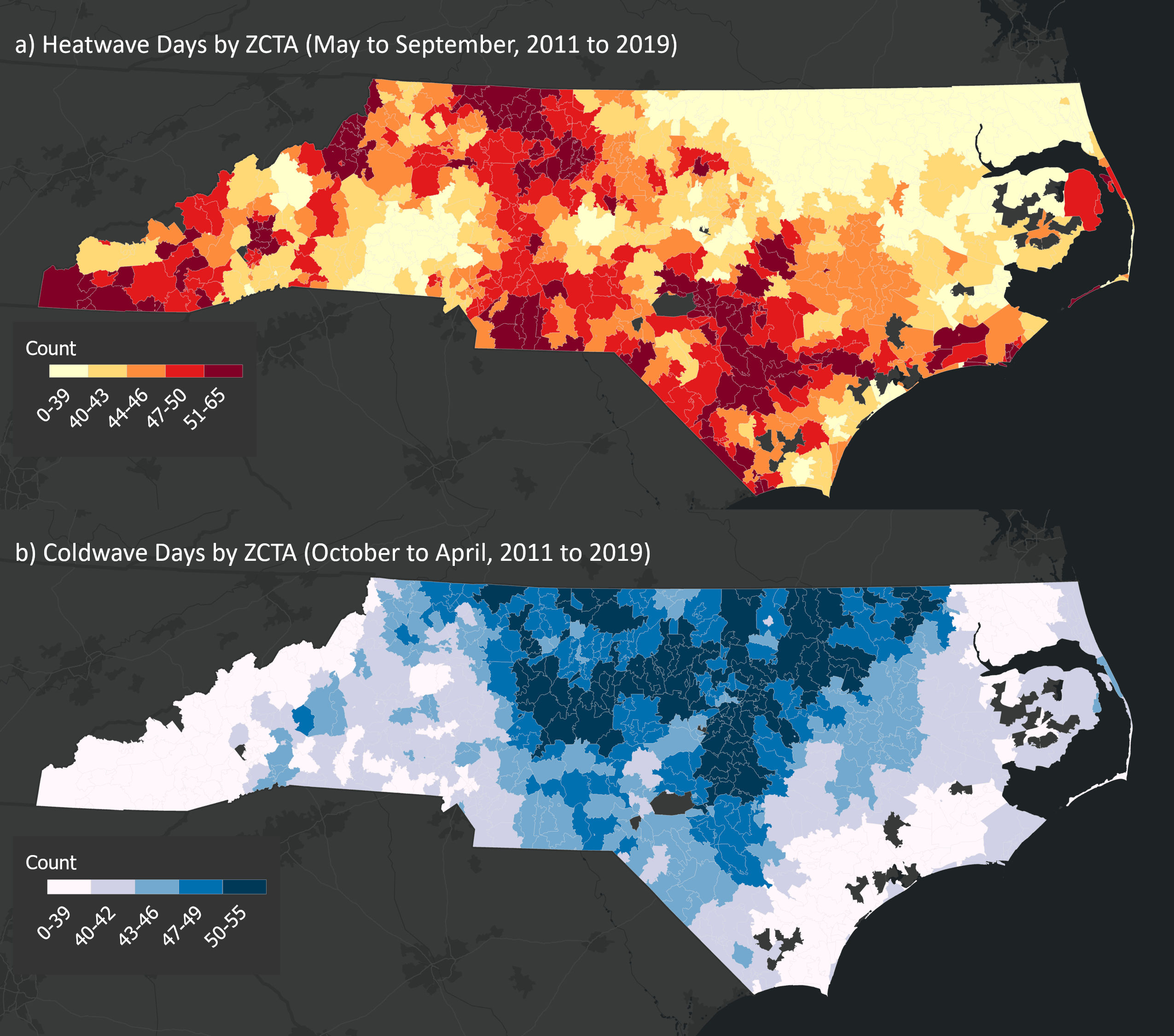

Supplement: Supplementary file 1 — Supplemental Figure 1. Count of heatwave and coldwave days by ZCTA (JPG 734 KB) [file 484_2025_3079_MOESM1_ESM.jpg]

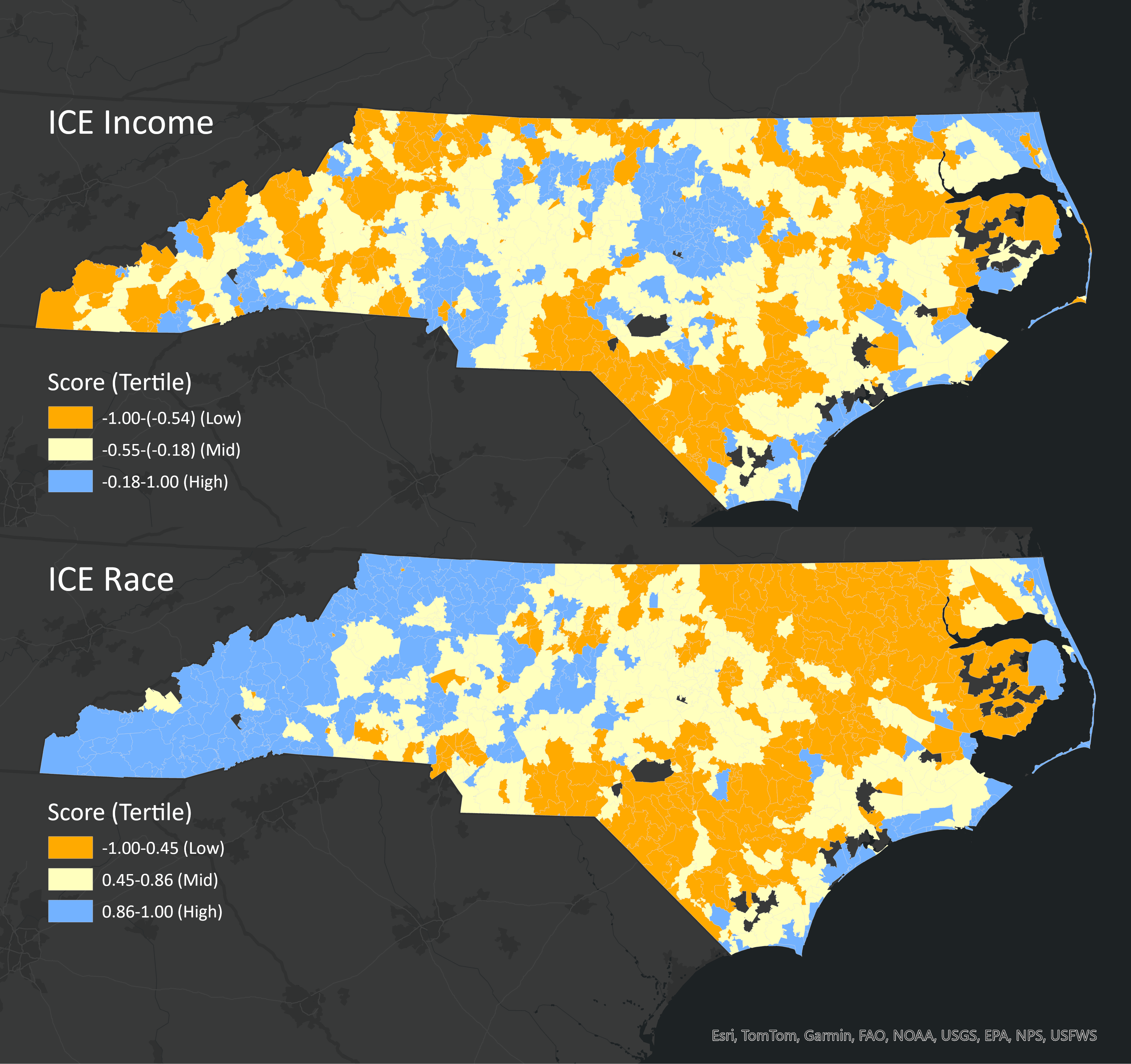

Supplement: Supplementary file 2 — Supplemental Figure 2. Economic segregation (ICE-E) and residential racial segregation (ICER) tertiles at the ZCTA level in North Carolina using data from the 2018 American Community Survey with 5-year estimates (PNG 1.76 MB) [file 484_2025_3079_MOESM2_ESM.png]

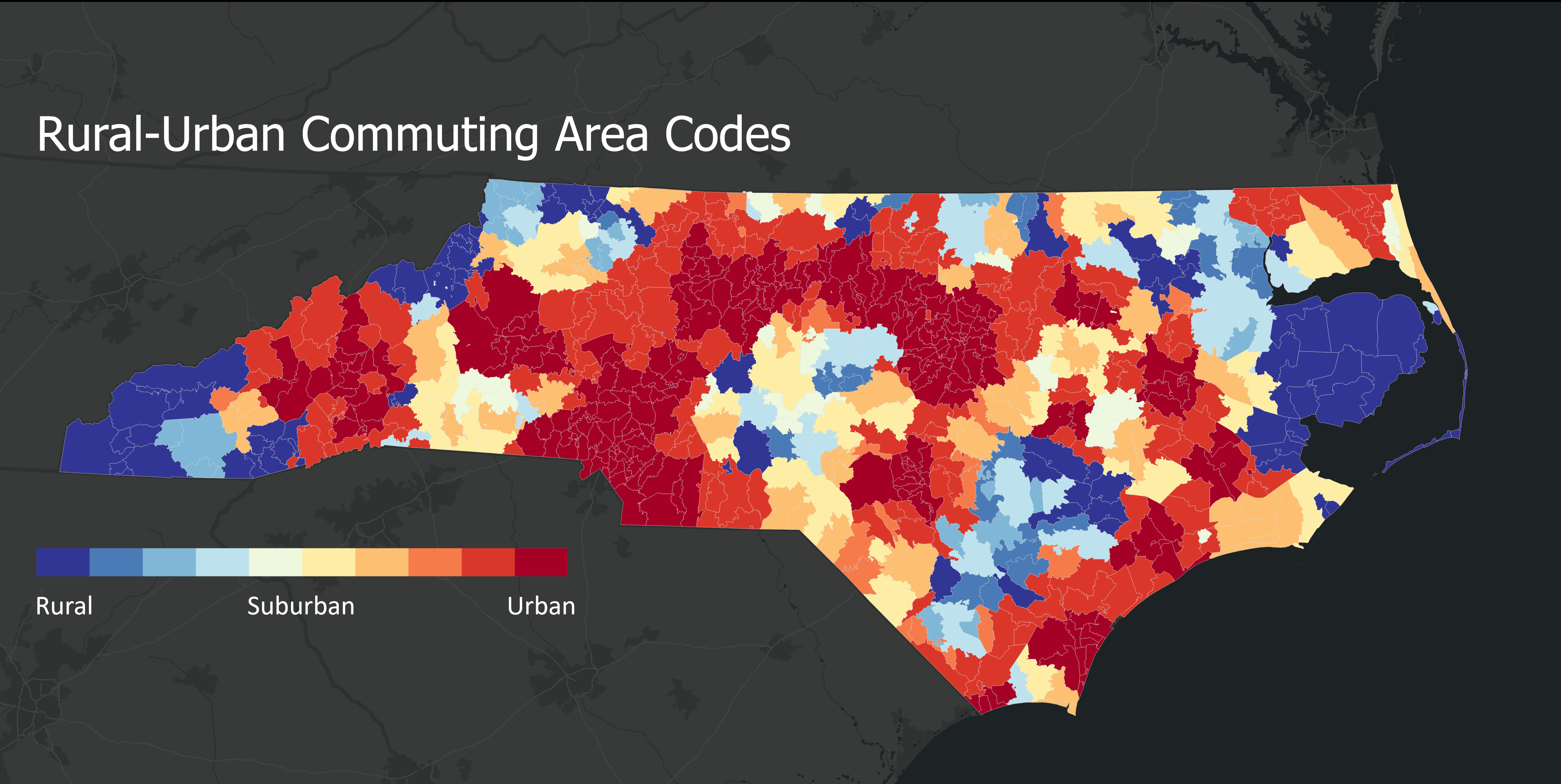

Supplement: Supplementary file 3 — Supplemental Figure 3. Distribution of Rural-Urban Commuting Area (RUCA) codes at the 2010 zip code tabulation area (ZCTA) level for North Carolina (PNG 1.25 MB) [file 484_2025_3079_MOESM3_ESM.png]

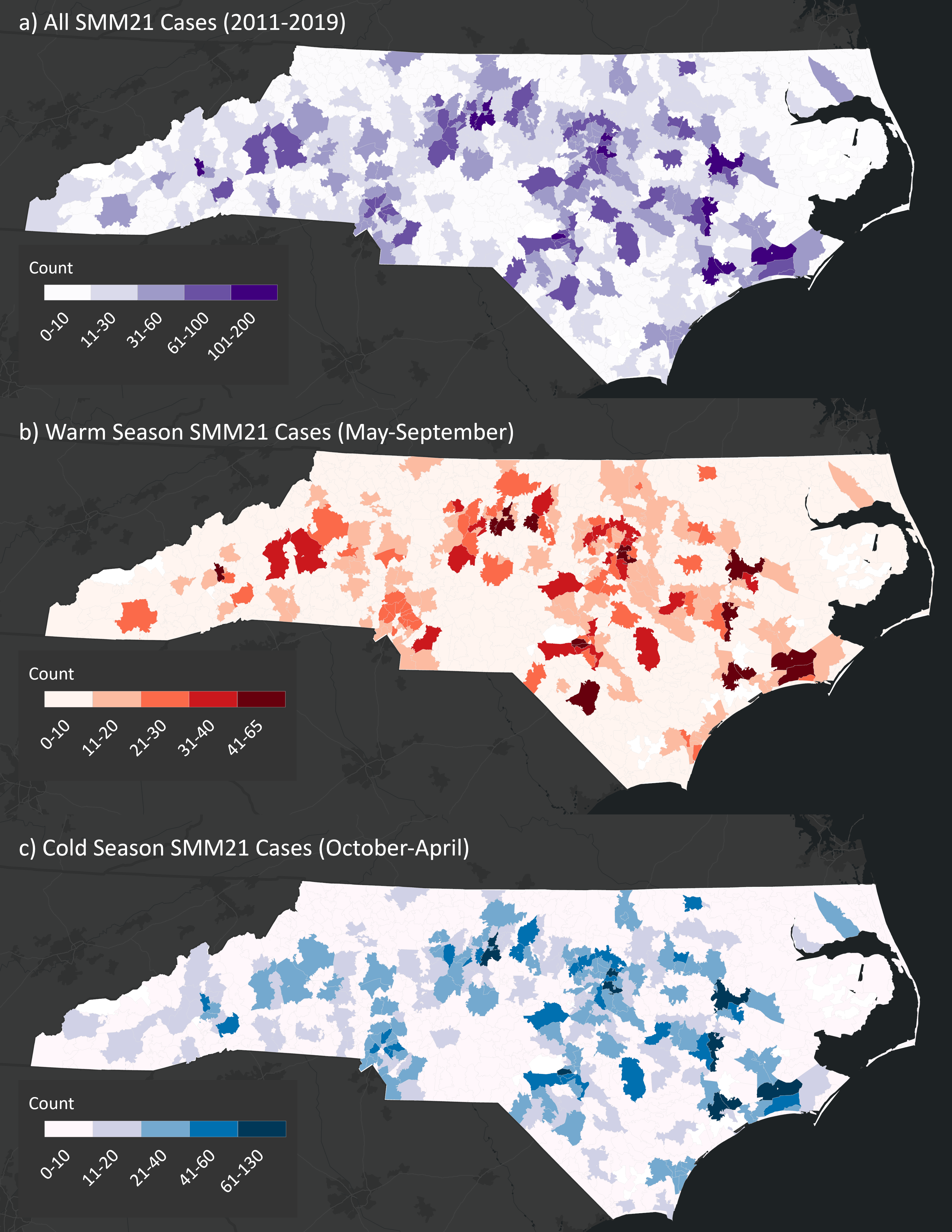

Supplement: Supplementary file 4 — Supplemental Figure 4. The total number of cases of severe maternal morbidity with blood transfusion (SMM21) for each ZCTA during the study period (2011 to 2019), warm season (May to September, 2011-2019), and cold season (October to April, 2011 to 2019) (PNG 2.41 MB) [file 484_2025_3079_MOESM4_ESM.png]

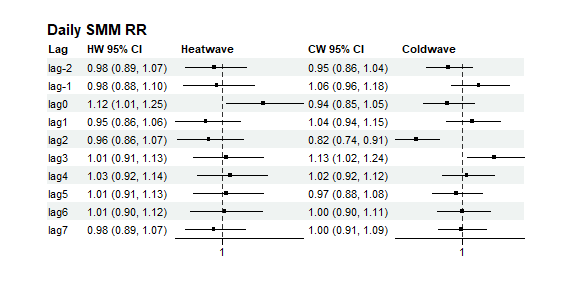

Supplement: Supplementary file 5 — Supplemental Figure 5. Daily relative risk values for SMM21 during heatwave and coldwave days (PNG 6.46 KB) [file 484_2025_3079_MOESM5_ESM.png]

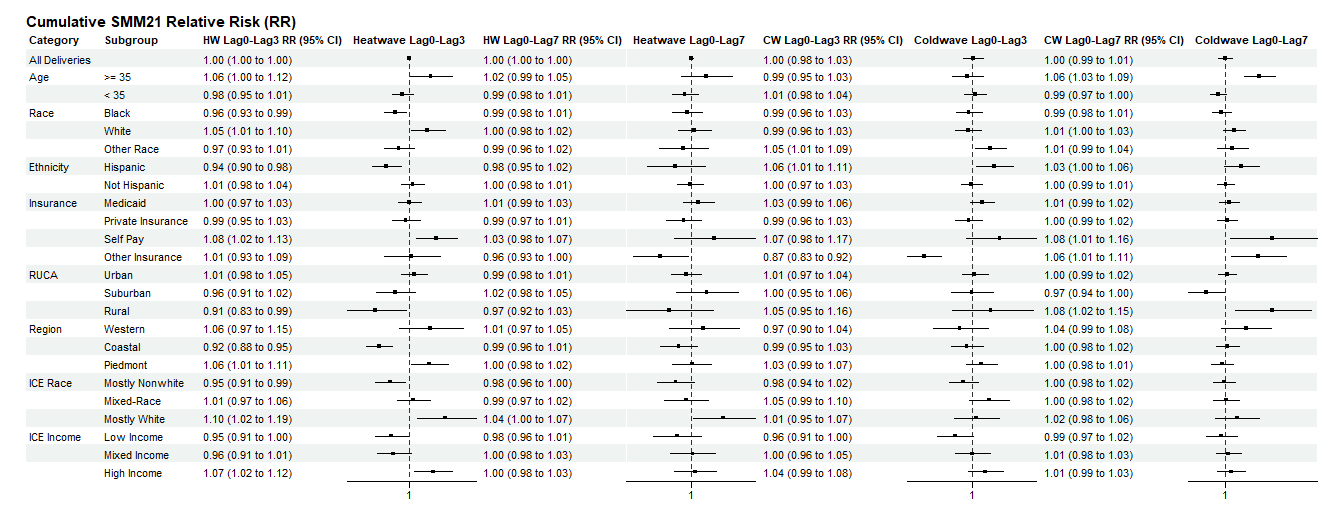

Supplement: Supplementary file 6 — Supplemental Figure 6. Cumulative relative risk (RR) values for SMM21 during lag0 to lag3 and lag0 to lag7 heatwave and coldwave periods (PNG 28.2 KB) [file 484_2025_3079_MOESM6_ESM.png]
