# Supplementary material for: Temperature extremes and maternal health: differential risks of severe maternal morbidity during heatwaves and coldwaves in North Carolina
Source: Int J Biometeorol. 2026 Feb 2;70(2):45. doi: 10.1007/s00484-025-03079-z (PMC12864237; doi:10.1007/s00484-025-03079-z)
Supplement: Supplementary file 7 — (PDF 6.38 MB) [file 484_2025_3079_MOESM7_ESM.pdf]

Supplemental Tables

Supplemental Table 1. SMM Indicators

Variable Name	Measuring	Type	Outcomes
AFE	SMM Indicator #5 Amniotic Fluid Embolism	Num	1 = Present<br>0 = Not Present
ARF	SMM Indicator #3 Acute Renal Failure	Num	1 = Present<br>0 = Not Present
ATE	SMM Indicator #17 Air and thrombotic embolism	Num	1 = Present<br>0 = Not Present
Aneurysm	SMM Indicator #2 Aneurysm	Num	1 = Present<br>0 = Not Present
Blood	SMM Indicator #18 Blood Transfusions	Num	1 = Present<br>0 = Not Present
CCR	SMM Indicator #7 Conversion of cardiac rhythm	Num	1 = Present<br>0 = Not Present
DIC	SMM Indicator #8 Disseminated intravascular coagulation	Num	1 = Present<br>0 = Not Present
Eclamp	SMM Indicator #9 Eclampsia	Num	1 = Present<br>0 = Not Present
Edema	SMM Indicator #12 Pulmonary Edema / acute heart failure	Num	1 = Present<br>0 = Not Present
HF	SMM Indicator #10 Heart failure / arrest during surgery or procedure	Num	1 = Present<br>0 = Not Present
Hyst	SMM Indicator #19 Hysterectomy	Num	1 = Present<br>0 = Not Present
MI	SMM Indicator #1 Acute myocardial infarction	Num	1 = Present<br>0 = Not Present
PCD	SMM Indicator #11 Puerperal cerebrovascular disorders	Num	1 = Present<br>0 = Not Present
Resp	SMM Indicator #4 Adult Respiratory distress syndrome	Num	1 = Present<br>0 = Not Present
SAC	SMM Indicator #13 Severe anesthesia complications	Num	1 = Present<br>0 = Not Present

SMM20	Composite SMM diagnosis derived from 20 indicators flagged with other codes (excluding Blood Transfusions)	Num	1 = Present<br>0 = Not Present
SMM21	Composite SMM diagnosis derived from 21 indicators flagged with other codes	Num	1 = Present<br>0 = Not Present
Sepsis	SMM Indicator #14 Sepsis	Num	1 = Present<br>0 = Not Present
Shock	SMM Indicator #15	Num	1 = Present<br>0 = Not Present
Sickle	SMM Indicator #16 Sickle cell disease with crisis	Num	1 = Present<br>0 = Not Present
Trach	SMM Indicator #20 Tracheostomy	Num	1 = Present<br>0 = Not Present
Vent	SMM Indicator #21 Ventilation	Num	1 = Present<br>0 = Not Present
VFib	SMM Indicator #6 Cardia arrest / ventricular fibrillation	Num	1 = Present<br>0 = Not Present

Supplemental Table 2. Case counts during heatwave and coldwave days, heatwave and coldwave periods, and matched unexposed periods for severe maternal morbidity without blood transfusion (SMM20) deliveries in North Carolina from 2011 to 2019, stratified by subgroup.

Group	Subpopulation	Heatwave Days (Lag0)	Heatwave Periods (Lag0 to Lag7)	Non-Heatwave Matched Periods	Coldwave Days (Lag0)	Coldwave Periods (Lag0 to Lag7)	Non-Coldwave Matched Periods
All		147	463	2027	212	650	1874
Age	>= 35	50	101	374	52	139	372
	< 35	97	366	1659	160	513	1505
Race	Black	54	151	678	57	212	623
	White	67	235	988	110	303	900
	Other	15	61	321	35	117	315
Ethnicity	Hispanic	11	51	204	19	65	199
	Non-Hispanic	136	407	1772	188	560	1635
Insurance	Medicaid	70	221	1014	102	324	963
	Private Ins	66	211	845	102	271	746
	Self-Pay	0	12	31	2	16	39
	Other Ins	11	23	143	6	40	135
RUCA	Urban	106	353	1521	177	539	1426
	Suburban	32	77	357	21	81	326
	Rural	9	33	149	14	30	122
Geographic Region	Western	17	59	278	15	62	227
	Coastal	32	143	701	54	197	656
	Piedmont	98	261	1048	143	391	991
ICE Income Tertile	Low (Mostly Low-Income)	33	100	437	40	125	382
	Mid (Mixed-Income)	49	161	827	51	205	738
	High (Mostly High-Income)	65	202	763	121	320	754
ICE Race Tertile	Low (Mostly Non-White)	84	204	850	81	275	889
	Mid (Mixed-Race)	46	185	831	96	279	717
	High (Mostly White)	17	74	346	35	96	268

Supplemental Table 3. Daily relative risk (RR) and 95% confidence interval (CI) estimates for SMM20 during heatwave and coldwave lag periods.

Lag	Heatwave SMM20		Coldwave SMM20	
	RR	CI	RR	CI
-2	1.00	0.99-1.01	0.93	0.81-1.06
-1	0.99	0.98-1.01	1.05	0.90-1.21
0	1.00	0.99-1.01	1.11	0.97-1.28
1	1.01	0.99-1.02	0.83	0.72-0.96
2	0.99	0.98-1.00	0.97	0.84-1.12
3	1.00	0.99-1.01	1	0.87-1.15
4	1.00	0.99-1.01	0.97	0.85-1.12
5	1.01	1.00-1.02	0.96	0.84-1.11
6	1.01	1.00-1.03	1.01	0.87-1.17
7	0.99	0.98-1.00	0.96	0.84-1.09

RR=Relative risk; CI=Confidence Interval
